# Exploring the potential mechanisms of OSBPL3 in metabolic dysfunction-associated steatotic liver disease by integrating bulk and single-cell RNA sequencing data

**DOI:** 10.1097/MD.0000000000046273

**Published:** 2025-12-26

**Authors:** Qianqian Wang, Chaoyu Zhu, Yuanyuan Xiao, Xinyi Wang, Wenjing Song, Shouxia Li, Fusong Jiang, Li Wei, Fei Hua

**Affiliations:** a Department of Endocrinology, The Third Affiliated Hospital of Soochow University, Changzhou, China; b Department of Endocrinology, Shanghai Sixth People’s Hospital Affiliated to Shanghai Jiao Tong University School of Medicine, Shanghai, China.

**Keywords:** macrophages, MASLD, monocytes, OSBPL3, single-cell RNA sequencing

## Abstract

OSBPL3 is vital for fatty liver disease, but its immune mechanisms in metabolic dysfunction-associated steatotic liver disease (MASLD) are unclear. This study investigates these mechanisms for MASLD treatment insights. MASLD datasets from public databases were used. OSBPL3 expression was analyzed by *t*-test in GSE57425, and its function explored via GSEA. Key cell types were identified in GSE129516 by scRNA-seq, followed by cell-to-cell communication and pseudo-temporal analyses. OSBPL3 expression differed significantly between high-fat and normal diet groups (*P* = .00048). It was enriched in the “oxidative phosphorylation” pathway, hinting at its role in energy metabolism and mitochondrial function in MASLD. Thirteen cell types were identified, with macrophages and monocytes as key types due to expression and cell percentage differences. Macrophages showed closer communication with granulocytes, fibroblasts, and erythrocytes in nonalcoholic steatohepatitis (NASH) diet samples. Macrophage and monocyte differentiation had 9 distinct states, with OSBPL3 highly expressed during metaphase. This study identified macrophages and monocytes as key cell types in OSBPL3’s mechanism in MASLD, offering valuable insights for targeted therapies.

## 1. Introduction

Metabolic dysfunction-associated steatotic liver disease (MASLD) refers to hepatic steatosis in patients with at least 1 metabolic risk factor such as obesity, diabetes, dyslipidemia and hypertension. MASLD can progress to cirrhosis and is an important cause of cryptogenic cirrhosis, which is one of the most ordinary liver diseases all over the world. There is a consensus that MASLD should be preferred to NAFLD.^[1,2]^ More than 25 percent of the world’s population is affected by MASLD. With the global epidemics of obesity and diabetes, MASLD is becoming a rapidly evolving health challenge.^[3]^ MASLD is a multifactorial disease with complex pathophysiological mechanisms including insulin resistance, activation of the neuroendocrine system, systemic inflammation, oxidative stress, dysregulation of the gut microbiome and increased ectopic fat. Current treatments for MASLD^[4]^ include the application of glucagon-like peptide-1 receptor agonists (GLP-1RA), dietary modification, weight loss, reduction of alcohol consumption, and reduction of metabolic risk factors, but no drugs have been approved specifically for MASLD. Therefore, there is an urgent need to explore the pathogenesis of MASLD from a biological perspective of biology in order to provide more accurate and optimized treatment for MASLD, so as to improve the symptoms of MASLD patients and reduce the risk of complications.

Oxysterol-binding protein-like protein 3 (OSBPL3) belongs to the oxysterol-binding protein (OSBP) family, which has 12 members (OSBP, OSBPL1-OSBPL11).^[5]^ OSBPL3 is a group of intracellular lipid receptors, which are expressed in different degrees in the endoplasmic reticulum and plasma membrane of human cells, and has been shown to participate in physiological processes such as lipid metabolism and signal transduction.^[6–9]^ Studies^[10]^ have shown that OSBPL3 plays a vital role in the occurrence and development of fatty liver disease, and that OSBPL3 is highly expressed in the liver of mice mutant for the glycation-deficient liver receptor homolog 1 (LRH-1) In addition, OSBPL3 enhances sterol regulatory element aggregation protein 1 (SREBP1) processing and promotes fat accumulation in the liver by activating de novo adipogenesis.^[11]^ Therefore, mRNA levels of OSBPL3 may be a diagnostic biomarker that can better stratify MASLD patients. However, there are no studies on the biological mechanism of OSBPL3 in MASLD.

Single-cell RNA sequencing (scRNA-seq) technology is a method for investigating gene expression patterns in Single cells to better understand the transcriptome, genome, proteome, epigenome and metabolome information of individual cells. In recent years, the emergence and advancement of scRNA-seq will provide potential possibilities for the discovery of previously potential disease-associated cell populations, cell functional states, and potential signaling regulators.^[12]^

In this study, public data sets were used to analyze the biological pathways, expression levels, transcription factor regulation and biological functions of OSBPL3 in MASLD. In addition, single-cell data sets were used to explore the communication network between OSBPL3-related key cells and other cells, as well as the pseudo-temporal differentiation process of key cells. Finally, the mechanism of OSBPL3 in MASLD was elucidated to provide new theoretical support and reference for the treatment and development of MASLD.

## 2. Materials and methods

### 2.1. Data collection

In our study, datasets related to MASLD were sourced from the gene expression omnibus database (https://www.ncbi.nlm.nih.gov/geo/). Specifically, bulk RNA sequencing (bulk RNA-seq) data were got from the GSE57425 dataset (GPL1261 platform), including hepatic tissue samples from 3 mice on a high-fat diet (case group) and 3 mice on a normal diet (control group). Additionally, scRNA-seq data were retrieved from the GSE129516 dataset (GPL21103 platform), comprising non-parenchymal cells from 3 mice on a NASH diet (case group) and 3 mice on a chow diet (control group).

### 2.2. Expression and function analyses of OSBPL3

Studies have shown that Oxysterol-binding protein-like 3 (OSBPL3) plays a key role in the development of fatty liver disease.^[10]^ In this study, the expression of OSBPL3 in high-fat diet and normal diet samples from the GSE57425 dataset was assessed using *t*-tests, including both 2-sample *t*-test and paired *t*-test (*P* <.05).

To explore potential interactions and functional relationships between OSBPL3 and other genes, the GeneMANIA database (http://genemania.org/) was used to identify associated genes and construct a gene-gene interaction network. Furthermore, to better understand the biological functions and pathways involving OSBPL3 in MASLD, functional enrichment analysis was conducted. First, in the GSE57425 dataset, Spearman correlations between OSBPL3 and other genes were calculated and ranked (from high to low) using the “psych” package (v 2.1.6) [https://CRAN.R-project.org/package=psych]. Then, the “CP: KEGG” gene set (species: mouse) was downloaded from MSigDB (https://www.gsea-msigdb.org/gsea/msigdb/) to serve as the background gene set. We subsequently performed GSEA using the “clusterProfiler” package (v 4.2.2),^[13]^ with a significance threshold of adj. *P* <.05, |NES| >1, and FDR <0.25. The results of GSEA were visualized using “enrichplot” package (v 1.18.3).^[14]^

### 2.3. Regulatory network analysis of OSBPL3

Understanding the regulatory mechanisms of OSBPL3 is crucial for unraveling its role in the occurrence and development of MASLD and other associated disorders. In our study, several databases were utilized to predict the miRNAs and lncRNAs involved in OSBPL3 regulation. Firstly, the miRTarBase database (https://awi.cuhk.edu.cn/~miRTarBase/miRTarBase_2025/php/index.php), TarBase database (http://www.microrna.gr/tarbase), and miRcode database were consulted to predict miRNAs that targeted OSBPL3. The key miRNAs were then identified by using the “VennDiagram” package (v 1.7.3) to overlap the predicted miRNAs from these 3 databases.^[15]^ Next, lncRNAs that potentially interact with these key miRNAs were identified using the miRnet database (https://www.mirnet.ca/). After organizing these interactions, a lncRNA-miRNA-mRNA network was constructed and visualized to further understand the complex regulatory relationships involving OSBPL3.

### 2.4. The scRNA-seq analysis

The scRNA-seq analysis was executed in the GSE129516 dataset applying the “Seurat” package (v 4.3.0).^[16]^ Quality control was initially performed to filter out low-quality cells and genes using the PercentageFeatureSet function. Cells with fewer than 200 detected genes were excluded. Genes expressed in fewer than 3 cells were excluded. Further, cells were retained if they met the following thresholds: 200 <nFeature_RNA (genes per cell) <7000, 200 <nCount_RNA (total RNA count per cell), and percent.mt (proportion of mitochondrial gene expression) <20%. After quality control, the data were normalized using the NormalizeData function. Next, highly variable genes (HVGs) were identified using the FindVariableFeatures function, and the results were visualized with the LabelPoints function. Principal component analysis (PCA) was performed using the RunPCA function on the HVGs, after data scaling with the ScaleData function. Principal components (PCs) were selected based on the JackStraw and the ElbowPlot functions (*P* <.05). To visualize the data following clustering, UMAP was applied with a resolution of 0.4. The marker genes of each cluster were identified using the FindAllMarkers function (|log2 fold change| >1, min.pct >0.1, FDR <0.05). The cell types were annotated using the “SingleR” package (v 2.2.0).^[17]^ The distributions of annotated cell types across all samples in the GSE129516 dataset were visualized. The *t*-test was applied to analyze the differences in the abundance of annotated cells between the NASH diet (case group) and chow diet (control group) samples, with significantly different cell types identified (*P* <.05). Subsequently, the “ReactomeGSA” package (v 1.16.1)^[18]^ was employed to perform functional enrichment analysis on the significantly differentially abundant cell types. Additionally, the expression of OSBPL3 in annotated cells was visualized using a UMAP plot and bubble diagram. The expression differences of OSBPL3 between the NASH diet and chow diet samples were analyzed in the whole scRNA-seq dataset GSE129516, as well as within each annotated cell type (*P* <.05). The cell clusters exhibiting differential expression of OSBPL3 and belonging to the significantly differentially abundant cell types were identified as key cell clusters associated with MASLD.

### 2.5. Cell-to-cell communication, heterogeneity, and pseudo-temporal analyses

To gain deeper insights into the annotated cell clusters, cell-to-cell communication analysis was performed on NASH diet and chow diet samples utilizing the “CellChat” package (v 1.6.1).^[19]^ The resulting cell-cell communication networks were visualized, highlighting the number and strength of interactions. Receptor-ligand pairs involved in intercellular interactions were also displayed.

Furthermore, secondary dimensional clustering analysis was conducted on the key cell types, with the appropriate PCs selected for further investigation. These key cell types were then divided into differential subclusters. Finally, to explore the differentiation states and trajectories of the key cell types, pseudo-temporal analysis was carried out utilizing the “Monocle” package (v 2.26.0).^[20]^ The expression dynamics of OSBPL3 throughout the pseudo-temporal progression were also analyzed.

### 2.6. Statistical analysis

Statistical analyses were clearly conducted applying R (v 4.3.4). Differences between groups were assessed applying the Wilcoxon test (*P* <.05). Notably, **** represented *P* <.0001, *** represented *P* <.001, ** represented *P* <.01, * represented *P* <.05, and ns represented *P* >.05 (no significant differences).

## 3. Results

### 3.1. Investigation the expression, function, and regulatory mechanism of OSBPL3

**Figure 1.**
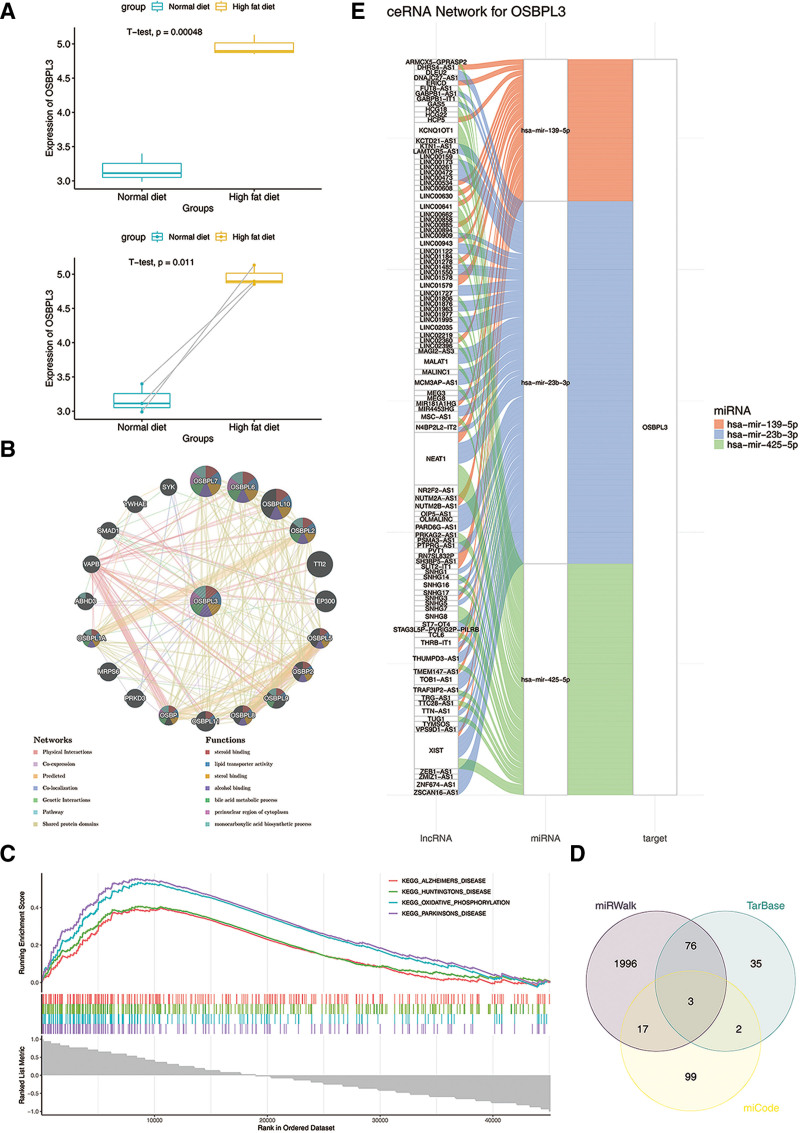
Investigation the expression, function, and regulatory mechanism of OSBPL3. (A) Distribution of OSBPL3 expression in the normal diet group and the high-fat diet group; (B) OSBPL3 related GGI network; (C) gene set enrichment analysis; (D) regulation of miRNA of OSBPL3; (e) ceRNA regulatory network of OSBPL3 gene. GGI = gene-gene interaction.

Differential expression analysis revealed that the expression of OSBPL3 was notably higher in high-fat diet samples from the GSE57425 dataset (*P* = .00048 in the 2-sample *t*-test and *P* = .011 in the paired *t*-test) (Fig. 1A). These findings suggested that OSBPL3 might have an important effect in the metabolic changes induced by a high-fat diet, potentially contributing to the occurrence and development of MASLD.

Moreover, using GeneMANIA, 20 genes functionally associated with OSBPL3 were identified. These included OSBPL7, OSBPL6, and OSBPL10, which were involved in critical processes such as “steroid binding” and “lipid transporter activity”(Fig. 1B). Additionally, GSEA revealed that OSBPL3 was notably enriched in pathways related to “Alzheimers disease,” “Huntingtons disease,” “oxidative phosphorylation,” and “Parkinsons disease” (Fig. 1C). These findings suggested that OSBPL3 might be involved in neurodegenerative diseases and cellular energy metabolism, highlighting its potential role in both lipid metabolism and neurodegenerative pathophysiology.

Finally, to investigate the potential regulatory mechanisms of OSBPL3, we predicted its regulatory factors. Specifically, 3 key miRNAs (hsa-miR-139-5p, hsa-miR-23b-3p, and hsa-miR-425-5p) were identified as potential regulators of OSBPL3 by overlapping the results from 3 independent databases (Fig. 1D). Additionally, lncRNAs targeting these key miRNAs were predicted, with NEAT1 identified as a potential co-regulator of all 3 miRNAs (Fig. 1E). These findings provided insight into the complex regulatory network involving OSBPL3, suggesting that miRNAs and lncRNAs might play important roles in modulating its expression.

### 3.2. Identification of 13 annotated cell types

**Figure 2.**
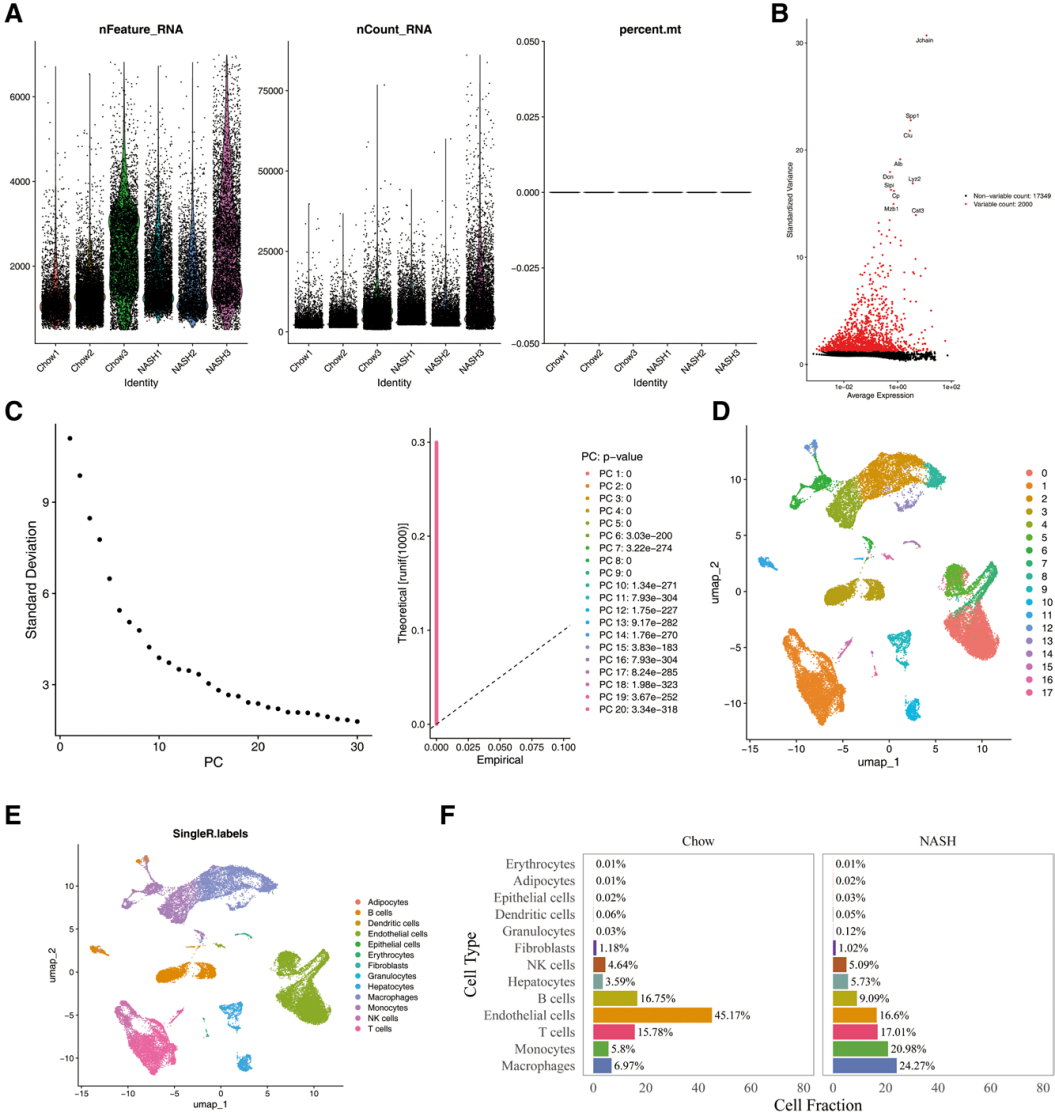
Identification of 13 annotated cell types. (A) After data quality control, nFeature_RNA, nCount_RNA, and percent.mt distribution maps; (B) high variant gene screening; (C) PCA elbow plot and PCA score and standard error diagram; (D) different cell subpopulations uniform manifold approximation and projection; (E) UMAP embedding map shows the distribution of various types of cells in different samples; (F) the largest cell population in chow diet samples and NASH diet samples. PCA = principal component analysis

Initially, 33,124 cells and 19,349 genes were retained after filtering out ineligible cells and genes in GSE129516 dataset (Fig. 2A). A total of 2000 HVGs were identified, with the top 10 HVGs highlighted (Fig. 2B). PCA was performed, selecting the top 20 PCs for further analysis (*P* <.05) (Fig. 2C). Using UMAP, the cells were classified into 18 distinct clusters (resolution = 0.4) (Fig. 2D). These clusters were subsequently annotated as adipocytes, B cells, epithelial cells, erythrocytes, macrophages, fibroblasts, dendritic cells, endothelial cells, granulocytes, hepatocytes, monocytes, NK cells, and T cells (Fig. 2E). Notably, endothelial cells constituted the largest cell population in chow diet samples (45.17%), while macrophages had the largest cell population in NASH diet samples (24.27%) (Fig. 2F).

### 3.3. Detection of macrophages and monocytes as key cell types of MASLD

**Figure 3.**
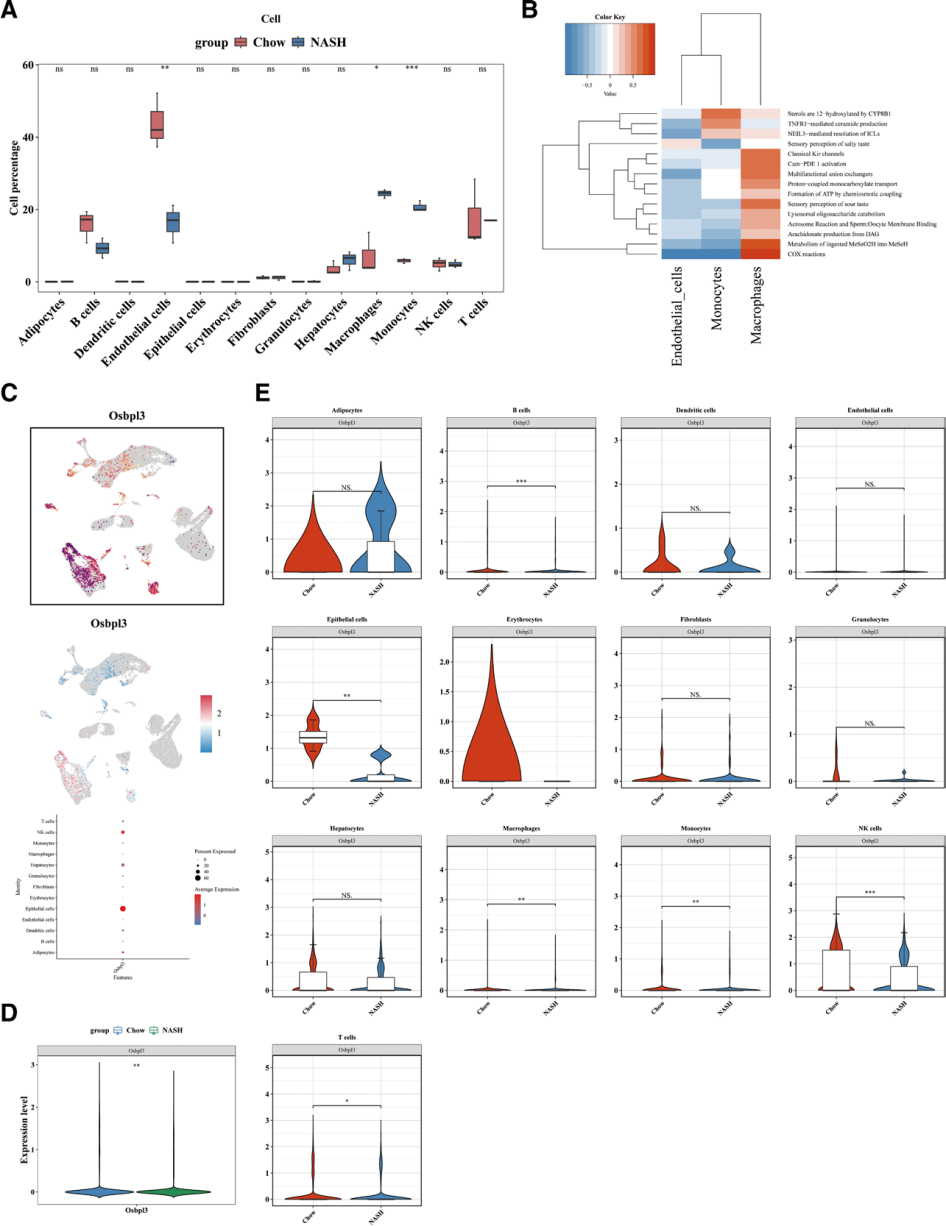
Detection of macrophages and monocytes as key cell types of MASLD. (A) The *t*-test revealed significant differences in the cell percentages of endothelial cells, macrophages, and monocytes between the NASH diet and chow diet samples; (B) These cell types were therefore considered significantly differentially abundant; (C) The expression of OSBPL3 in each annotated cell type; (D) The different expression of OSBPL3 between the NASH diet and chow diet samples in the GSE129516 dataset; (E) OSBPL3 different expression between the 2 groups in B cells, epithelial cells, macrophages, monocytes, NK cells, and T cells. MASLD = metabolic dysfunction-associated steatotic liver disease.

The t test revealed significant differences in the cell percentages of endothelial cells, macrophages, and monocytes between the NASH diet and chow diet samples (*P* <.05) (Fig. 3A). These cell types were therefore considered significantly differentially abundant. Further analysis showed that monocytes were significantly enriched in the “Sterols are 12-hydroxylated by CYP8B1” pathway, while macrophages were notably enriched in pathways like “COX reactions” and “metabolism of ingested MeSeO2H into MeSeH” (Fig. 3B).

Moreover, the expression of OSBPL3 in each annotated cell type was examined (Fig. 3C). The expression of OSBPL3 showed significant differences between the NASH diet and chow diet samples in the GSE129516 dataset (Fig. 3D). Specifically, OSBPL3 expression was significantly different between the 2 groups in B cells, epithelial cells, macrophages, monocytes, NK cells, and T cells (*P* <.05) (Fig. 3E). Integrating these results, we identified macrophages and monocytes as key cell types in MASLD, as both showed significant differences in expression between the NASH and chow diet groups and were classified as differentially abundant cell types.

### 3.4. Exploration of intercellular communication and differentiation trajectories of key cell types

**Figure 4.**
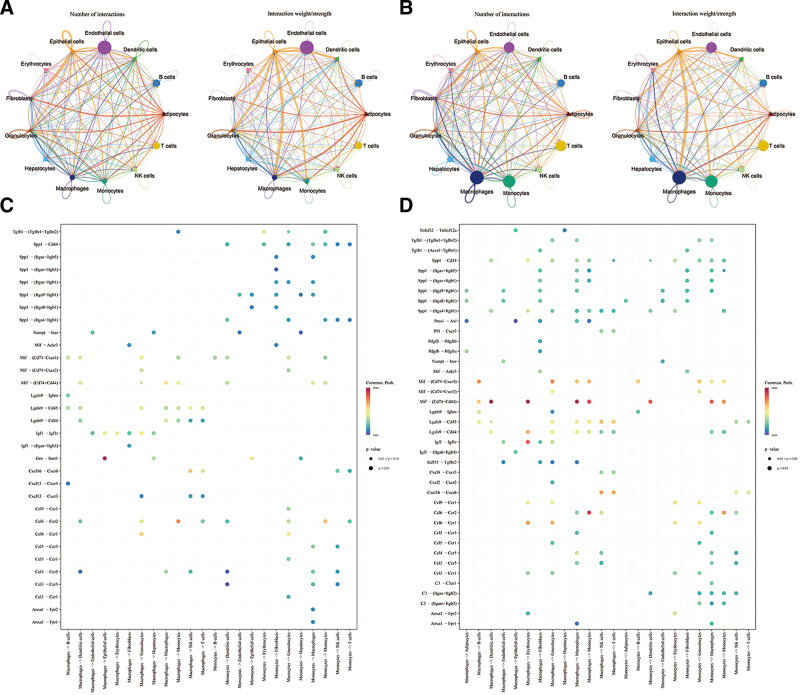
Exploration of intercellular communication and differentiation trajectories of key cell types. (A and B) Cell-to-cell communication analysis among annotated cells in both NASH diet and chow diet samples; (C and D) Communication probability of the MIF-(CD74 + CD44) ligand-receptor pair in NASH diet samples. NASH = nonalcoholic steatohepatitis.

Cell-to-cell communication analysis revealed frequent interactions among annotated cells in both NASH diet and chow diet samples. Notably, macrophages exhibited more intimate communication with granulocytes, fibroblasts, and erythrocytes in NASH diet samples compared with chow diet samples (Fig. 4A and B). Furthermore, the MIF-(CD74 + CD44) ligand-receptor pair demonstrated a high communication probability in NASH diet samples (Fig. 4C and D).

**Figure 5.**
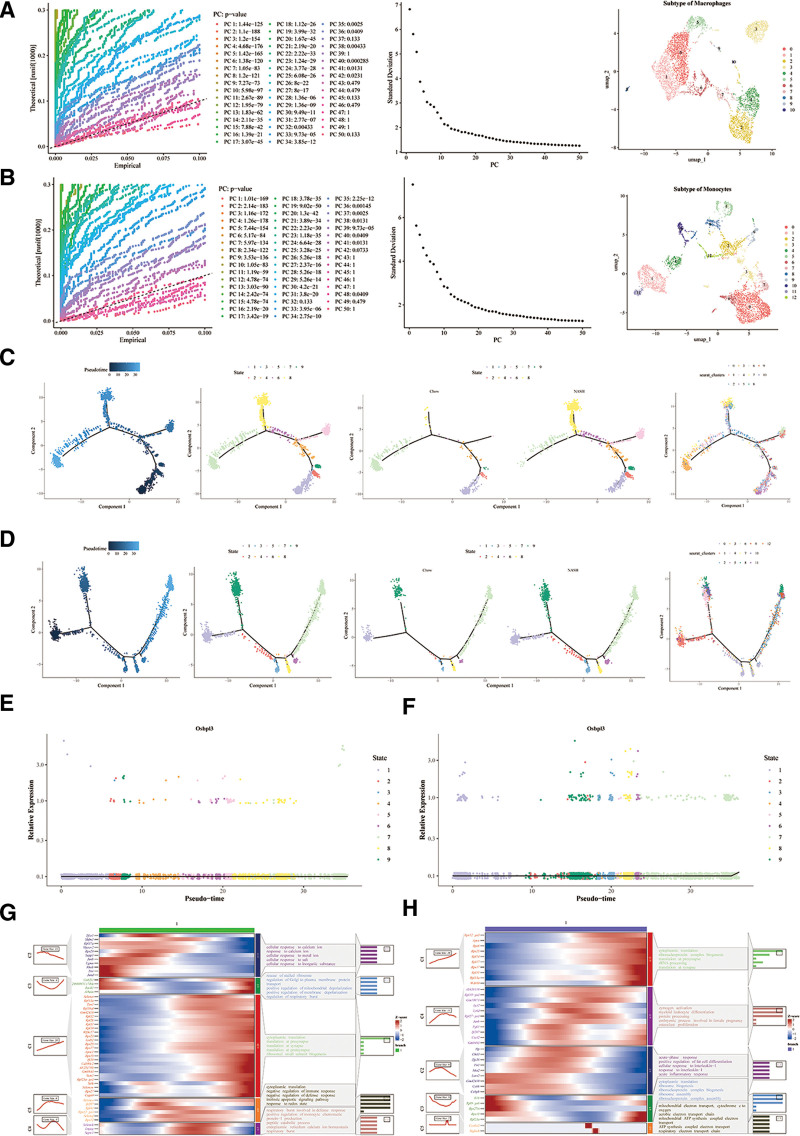
Exploration of intercellular communication and differentiation trajectories of key cell types. (A and B) Secondary dimensional clustering analysis identified subclusters of macrophages and monocytes; (C and D) Pseudo-temporal analysis of macrophage and monocyte different developmental states; (E and F) the expression of OSBPL3 during metaphase of macrophage and monocyte differentiation; (G and H) during macrophage and monocyte differentiation, genes with similar expression patterns were grouped into 5 clusters.

Secondary dimensional clustering analysis identified 11 subclusters of macrophages (Fig. 5A) and 13 subclusters of monocytes (Fig. 5B). Pseudo-temporal analysis of macrophage and monocyte differentiation revealed a progressive trajectory from dark blue to light blue, with distinct classifications into 9 developmental states (Fig. 5C and D). The expression of OSBPL3 was most concentrated during the metaphase of macrophage and monocyte differentiation (Fig. 5E and F). Additionally, Figure 5G and H illustrates that during macrophage and monocyte differentiation, genes with similar expression patterns were grouped into 5 clusters (C1–C5). Functions enriched in each cluster were biologically relevant.

In this study, based on MASLD in the gene expression omnibus database, we performed a series of bioinformatic analyses on the MASLD-related transcriptome dataset (GSE57425) and single-cell dataset (GSE129516). Specifically, first, in GSE57425, we evaluated the difference in OSBPL3 expression between MASLD group samples and control group samples to explore the relevant functions and pathways involved in OSBPL3 and the potential regulatory mechanisms involved in OSBPL3. Secondly, we annotated cell types in GSE129516, and further identified macrophages and monocytes as key cells by combining the difference in the abundance of annotated cells between NASH and control samples and the difference in the expression of OSBPL3 in each annotated cell. Finally, we conducted cell communication and quasi-temporal analysis to explore the communication between key cells and other cells and the differentiation process of key cells. These analyses provide new insights into the potential mechanism of OSBPL3 in MASLD and contribute to the treatment of MASLD.

## 4. Discussion

In recent years, the incidence of MASLD as the most prevalent chronic liver diseases has increased year by year, and threaten human health and increase the economic burden in the world.^[21–23]^ However, there is no specific treatment for the prevention or treatment of MASLD. Studies have found that in mice constructed with glycation-deficient liver receptor homolog 1 (LRH-1) mutation, the expression level of OSBPL3 in liver is significantly increased, which promotes the incidence and development of MASLD.^[10,11]^ This indicates that OSBPL3 is indeed involved in the incidence and development of MASLD and can participate in the treatment of MASLD disease progression. Our study found that there were distinctive differences in the expression level of the OSBPL3 gene between the MASLD group and the control group. OSBPL3 was significantly up-regulated in the MASLD group. Moreover, through analyzing the percentage differences in annotated cells and the expression differences of OSBPL3 between NASH diet (case group) and chow diet (control group) samples, macrophages and monocytes were identified as key cell types. Macrophages exhibited more intimate communication with granulocytes, fibroblasts, and erythrocytes in NASH diet samples compared to chow diet samples. Additionally, the expression of OSBPL3 was most concentrated during the metaphase of macrophage and monocyte differentiation. This study focused on the mechanism of OSBPL3 in MASLD, and identified macrophages and monocytes as the key cell types. This finding provides a theoretical basis/new insight for exploring the mechanism of MASLD and OSBPL3.

Daisuke Aibara et al found that^[10]^ OSBPL3 was highly expressed in humans and mouse fatty livers. Moreover, studies have shown that the upregulation expression of OSBPL3 in the liver was also observed in the fatty livers of ob/ob mice with type 2 diabetes, db/db mice with a leptin receptor mutation, and alcohol-fed mice.^[24,25]^ These studies are consistent with our findings of OSBPL3 expression trends. Functional enrichment analysis showed that OSBPL3 was significantly enriched in oxidative phosphorylation and other pathways. Yotaro et al^[26]^ found that oxidative stress plays a key role in liver tissue damage and in the occurrence and development of hepatocellular carcinoma (HCC). Loss of protein kinase C (PKC) in hepatocytes promotes autophagy and oxidative phosphorylation. It^[27]^ has also been reported that the fatty acid cargo of tumor EVPs, especially the commonly palmitic acid, created a microenvironment that promotes inflammation, inhibiting oxidative phosphorylation and fatty acid metabolism, playing an important role in the formation of fatty liver. In addition, microRNAs and long noncoding RNAs (lncRNAs) play a vial role in regulating metabolic processes in MASLD, and have become therapeutic targets for MASLD. Schutz et al^[28]^ found that the expression levels of miR-34a and miR-21 are increased in MASLD animal models, which promote the occurrence of MASLD. Another study^[29]^ found that in cervical cancer patients, the expression level of OSBPL3 decreased due to miR-497/195 targeting those genes, which affected their overall survival. Some scholars have also found that^[30]^ lncRNA FAM66C regulates downstream target genes to drive intrahepatic cholangiocarcinoma progression and glycolysis activity through sponge action of miR-23b-3p, suggesting that FAM66C may be a potential target for the treatment of ICC. Some miRNAs targeting OSBPL3 regulation are also consistent with our study.

Recently, more and more scholars are interested in the potential therapeutic value of immunometabolism in MASLD. In a recent study,^[31]^ macrophages were identified as key players in the progression of MASLD. Transgenic mice with specific MST1 and MST2 deletion in macrophages/monocytes after high-fat feeding induced MASLD models, found that liver inflammation and fibrosis are enhanced due to the absence of MST1/2 in liver macrophages in MASLD. These results indicate that liver macrophages have an important relationship with the progression of MASLD. Another study^[32]^ indicated that the expression of miR-204-3p was reduced in the macrophages and liver of mice and patients with MASLD. In MASLD patients, the level of miR-204-3p in peripheral blood mononuclear cells was negatively correlated with the severity of liver inflammation and injury. miR-204-3p inhibits macrophage inflammation, coordinates macrophage action on hepatocytes and HSCS, and ameliorates steatohepatitis. Macrophage miR-204-3p may be a therapeutic target for MASLD. Study by Sangineto et al^[33]^ finding that monocytes (Mos) play a critical role in the evolution of MASLD to metabolically dysfunctionally-associated steatohepatitis (MASH), analysis of the public scRNA-seq dataset confirmed that in a mouse model of MASH, liver Mo-derived macrophages are increased in the glycolytic energy pathway. A study^[34]^ found that macrophage-mediated inflammation is associated with the pathogenesis of MASH. The expression of β-arrestin 2 in liver macrophages and circulating monocytes was significantly increased in MASH patients and positively correlated with the severity of MASLD. Our study found that the expression level of OSBPL3 was relatively high in these 2 key cell differentiation stages, which further explored the mechanism of OSBPL3 in MASLD.

This study found that the expression of OSBPL3 was significantly different between the MASLD group and the control group. The enrichment of OSBPL3 in the oxidative phosphorylation pathway was investigated. At the single-cell level, macrophages and monocytes were identified as key cells of MASLD, and it was found that OSBPL3 was more highly expressed at the middle-differentiation stage of the 2 key cells through quasi-time series analysis. These analyses provide insights into the underlying mechanism of OSBPL3 in MASLD and inform theory-driven basis for the treatment of MASLD. In addition, this study also has limitations: there is a lack of more animal and cell experimental verification, such as gene knockout, animal modeling, etc. In addition, we will strengthen this part of the study in the future to more comprehensively reveal the potential mechanism of OSBPL3 in the treatment of MASLD more comprehensively, and provide more theoretical references for MASLD.

## 5. Conclusions

This study integrates bulk and single-cell RNA sequencing data to elucidate the potential mechanisms of OSBPL3 in MASLD. Results show that OSBPL3 is significantly upregulated in MASLD and enriched in critical pathways such as oxidative phosphorylation, indicating its involvement in energy metabolism and mitochondrial function. Single-cell analysis further identifies macrophages and monocytes as key cell types in MASLD, with significant differences in cell abundance and OSBPL3 expression between NASH and control groups. Additionally, macrophages exhibit more frequent communication with other cells in NASH samples, and OSBPL3 expression peaks during the metaphase of macrophage and monocyte differentiation. These findings provide new theoretical insights into the role of OSBPL3 in MASLD and offer potential cellular targets for targeted therapies.

## Acknowledgments

We would like to express our sincere gratitude to all individuals and organizations who supported and assisted us throughout this research. Special thanks to the following authors: Qianqian Wang, Chaoyu Zhu, Yuanyuan Xiao, Xinyi Wang, Wenjing Song, Shouxia Li, Fusong Jiang, Li Wei and Fei Hua. And thanks to the Fundamental Research Funds for the Central Universities and National Key R&D Program of China for the funding needed for this research. In conclusion, we extend our thanks to everyone who has supported and assisted us along the way. Without your support, this research would not have been possible.

## Author contributions

**Conceptualization:** Qianqian Wang, Fei Hua.

**Data curation:** Qianqian Wang.

**Formal analysis:** Qianqian Wang, Xinyi Wang.

**Funding acquisition:** Qianqian Wang, Fei Hua.

**Investigation:** Qianqian Wang, Fusong Jiang.

**Methodology:** Qianqian Wang, Chaoyu Zhu, Wenjin Song, Li Wei.

**Project administration:** Chaoyu Zhu, Yuanyuan Xiao, Wenjin Song, Shouxia Li.

**Resources:** Fusong Jiang.

**Software:** Yuanyuan Xiao, Shouxia Li.

**Supervision:** Chaoyu Zhu.

**Visualization:** Xinyi Wang, Li Wei.
